# Supplementary material for: Trapping liquids may bias the results of beetle diversity assessment
Source: PeerJ. 2023 Dec 8;11:e16531. doi: 10.7717/peerj.16531 (PMC10712302; doi:10.7717/peerj.16531)
Supplement: Data S1 [file peerj-11-16531-s001.docx]

**
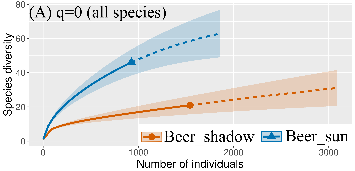

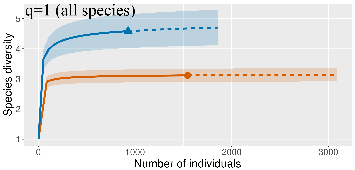
**
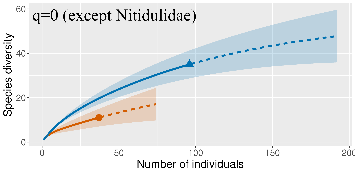

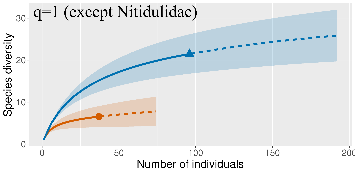


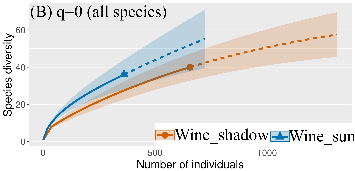

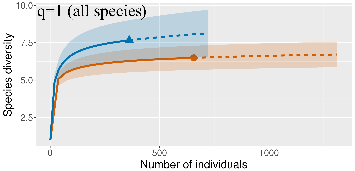

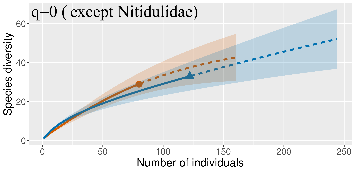

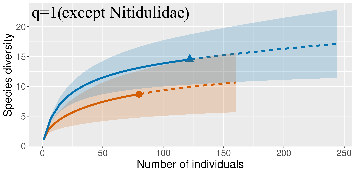


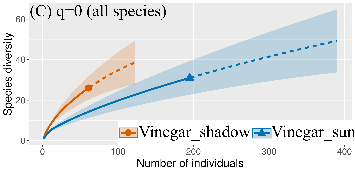
**
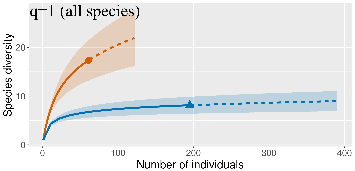

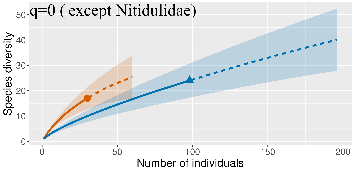

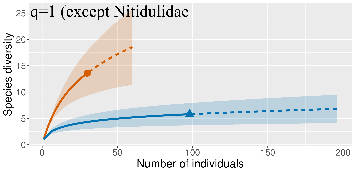
**


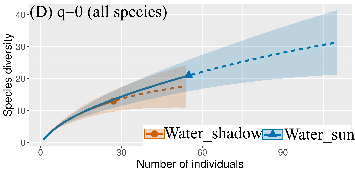
**
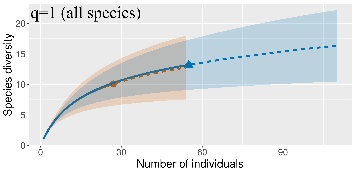
**
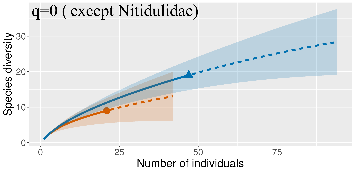

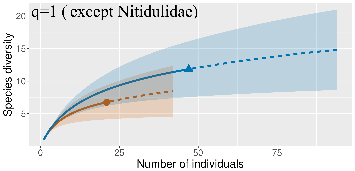


Figure 1S Sample-size-based rarefaction and extrapolation sampling gamma diversity curve showing Hill's numbers, abundance data. Distribution by individual baits and different exposure**,** q=0 (species richness) and q=1 (the exponential of Shannon’s entropy index) and influence to excluded families Nitidulidae. Colored shaded areas are the 95% confidence intervals. Solid symbols represent total number of species and extrapolation (dashed lines), up to double the reference sample size.

Tab 1S. Raw data: List of species that were recorded during the study. Alphabetical order of by families broken down by bait.

|  |  | vinegar | beer | wine | water | sum |  |
| --- | --- | --- | --- | --- | --- | --- | --- |
| Biphyllidae | |  |  | 1 |  | 1 |  |
|  | *Diplocoelus fagi* (Guérin-Ménéville, 1838) |  |  | 1 |  | 1 |  |
| Buprestidae | | 4 | 4 | 4 |  | 12 |  |
|  | *Agrilus angustulus* (Illiger, 1803) |  | 1 | 1 |  | 2 |  |
|  | *Agrilus hastulifer* (Ratzeburg, 1839) | 2 |  |  |  | 2 |  |
|  | *Agrilus obscuricollis* (Kiesenwetter, 1857) |  |  | 1 |  | 1 |  |
|  | *Agrilus viridis* (Linnaeus, 1758) |  |  | 1 |  | 1 |  |
|  | *Anthaxia fulgurans* (Schrank, 1787) |  |  | 1 |  | 1 |  |
|  | *Anthaxia quadripunctata* (Linnaeus, 1758) | 1 | 1 |  |  | 2 |  |
|  | *Chrysobothris affinis* (Fabricius, 1794) | 1 | 2 |  |  | 3 |  |
| Carabidae | | 1 | 12 | 8 |  | 21 |  |
|  | *Carabus hortensis* (Linnaeus, 1758) |  | 12 | 5 |  | 17 |  |
|  | *Carabus intricatus* (Linnaeus, 1761) | 1 |  | 1 |  | 2 |  |
|  | *Limodromus krynickii* (Sperk, 1835) |  |  | 2 |  | 2 |  |
| Cerambycidae | | 1 | 12 | 26 | 3 | 42 |  |
|  | *Alosterna tabacicolor* (DeGeer, 1775) |  |  |  | 1 | 1 |  |
|  | *Anaesthetis testacea* (Fabricius, 1781) |  |  | 3 |  | 3 |  |
|  | *Leiopus linnei* (Wallin, Nylander & Kvamme, 2009) | | 1 |  |  | 1 |  |
|  | *Leptura maculata* (Poda, 1761) |  | 3 | 4 |  | 7 |  |
|  | *Stictoleptura rubra* (Linnaeus, 1758) | 1 |  |  | 1 | 2 |  |
|  | *Phymatodes testaceus* (Linnaeus, 1758) |  | 1 | 2 |  | 3 |  |
|  | *Stenurella melanura* (Linnaeus, 1758) |  | 2 | 7 | 1 | 10 |  |
|  | *Xylotrechus antilope* (Schönherr, 1817) |  | 5 | 10 |  | 15 |  |
| Coccinellidae | |  |  | 1 |  | 1 |  |
|  | *Oenopia lyncea* (Olivier, 1808) |  |  | 1 |  | 1 |  |
| Curculionidae | | 12 | 8 | 14 | 25 | 59 |  |
|  | *Brachyderes incanus* (Linnaeus, 1758) | 1 | 1 | 5 | 5 | 12 |  |
|  | *Curculio pellitus* (Boheman, 1843) |  | 1 |  |  | 1 |  |
|  | *Dryocoetes villosus* (Fabricius, 1792) | 2 |  |  | 4 | 6 |  |
|  | *Gasterocercus depressirostris* (Fabricius, 1792) | | 1 | 1 |  | 2 |  |
|  | *Hylesinus oleiperda* (Fabricius, 1792) | 1 |  |  |  | 1 |  |
|  | *Magdalis fuscicornis* Desbrochers, 1870 | 1 |  |  |  | 1 |  |
|  | *Platypus* cylindrus (Fabricius, 1792) |  |  |  | 1 | 1 |  |
|  | *Scolytus intricatus* (Ratzeburg, 1837) | 4 | 1 | 1 | 6 | 12 |  |
|  | *Sitona lineatus* (Linnaeus, 1758) |  |  | 1 |  | 1 |  |
|  | *Strophosoma capitatum* (De Geer, 1775) |  |  | 2 |  | 2 |  |
|  | *Xyleborinus saxesenii* (Ratzeburg, 1837) |  | 2 |  |  | 2 |  |
|  | *Xyleborus dryographus* (Ratzeburg, 1837 | 3 | 2 | 4 | 9 | 18 |  |
| Dermestidae | |  |  |  | 1 | 1 |  |
|  | *Anthrenus verbasci* (Linnaeus, 1767) |  |  |  | 1 | 1 |  |
| Elateridae | | 3 | 19 | 44 | 3 | 69 |  |
|  | *Agriotes pilosellus* (Schönherr, 1817) |  |  | 1 |  | 1 |  |
|  | *Brachygonus megerlei* (Lacordaire, 1835) |  |  | 1 |  | 1 |  |
|  | *Cardiophorus nigerrimus* (Erichson, 1840) | 3 |  | 1 |  | 4 |  |
|  | *Melanotus crassicollis* (Erichson, 1841) |  | 16 | 39 | 2 | 57 |  |
|  | *Melanotus villosus* (Geoffroy in Fourcroy, 1785) | | 1 |  |  | 1 |  |
|  | *Melanotus castanipes* (Paykull, 1800) |  |  | 1 | 1 | 2 |  |
|  | *Nothodes parvulus* (Panzer, 1799) |  | 1 |  |  | 1 |  |
|  | *Prosternon tessellatum* (Linnaeus, 1758) |  | 1 | 1 |  | 2 |  |
| Erotylidae | |  |  | 1 |  | 1 |  |
|  | *Triplax lepida* (Faldermann, 1835) |  |  | 1 |  | 1 |  |
| Histeridae | |  |  | 1 |  | 1 |  |
|  | *Paromalus flavicornis* (Herbst, 1791) |  |  | 1 |  | 1 |  |
| Lampyridae | | 2 |  |  |  | 2 |  |
|  | *Lamprohiza splendidula* (Linnaeus, 1767) | 1 |  |  |  | 1 |  |
|  | *Lampyris noctiluca* (Linnaeus, 1767) | 1 |  |  |  | 1 |  |
| Leiodidae | | 1 |  |  |  | 1 |  |
|  | *Anisotoma humeralis* (Fabricius, 1792) | 1 |  |  |  | 1 |  |
| Lycidae | | 1 | 2 | 1 | 1 | 5 |  |
|  | *Lygistopterus sanguineus* (Linnaeus, 1758) | 1 | 2 | 1 | 1 | 5 |  |
| Melandryidae | |  |  | 1 |  | 1 |  |
|  | *Orchesia undulata* (Kraatz, 1853) |  |  | 1 |  | 1 |  |
| Melasidae | | 1 |  |  | 2 | 3 |  |
|  | *Dromaeolus barnabita* (A. & G.B. Villa, 1838) | 1 |  |  | 2 | 3 |  |
| Melyridae | | 5 | 3 | 2 | 2 | 12 |  |
|  | *Dasytes niger* (Linnaeus, 1761) |  | 2 |  |  | 2 |  |
|  | *Dasytes plumbeus* (O.F. Müller, 1776) | 5 | 1 | 2 | 2 | 10 |  |
| Monotomidae | |  | 1 | 2 |  | 3 |  |
|  | *Rhizophagus bipustulatus* (Fabricius, 1792) | | 1 |  |  | 1 |  |
|  | *Rhizophagus parvulus* (Paykull, 1800) |  |  | 2 |  | 2 |  |
| Mordellidae | | 83 | 12 | 7 | 16 | 118 |  |
|  | *Mordella aculeata* (Linnaeus, 1758) | 1 |  |  |  | 1 |  |
|  | *Mordella brachyura* (Mulsant, 1856) | 63 | 11 | 6 | 15 | 95 |  |
|  | *Mordella holomelaena* (Apfelbeck, 1914) | 9 |  | 1 |  | 10 |  |
|  | *Mordellistena neuwaldeggiana* (Panzer, 1796) | | 1 |  |  | 1 |  |
|  | *Tomoxia bucephala* (Costa, 1854) | 3 |  |  |  | 3 |  |
| Mycetophagidae | |  | 1 | 2 |  | 3 |  |
|  | *Litargus connexus* (Fourcroy, 1785) |  | 1 | 1 |  | 2 |  |
|  | *Triphyllus bicolor* (Fabricius, 1792) |  |  | 1 |  | 1 |  |
| Nitidulidae | | 128 | 2337 | 817 | 15 | 3297 |  |
|  | *Brassicogethes aeneus* (Fabricius, 1775) | 2 | 2 | 1 | 1 | 6 |  |
|  | *Brassicogethes subaeneus* (Sturm, 1845) | 1 | 1 |  |  | 2 |  |
|  | *Carpophilus hemipterus* (Linnaeus, 1758) |  | 1 |  |  | 1 |  |
|  | *Cryptarcha strigata* (Fabricius, 1787) | 52 | 1448 | 318 | 9 | 1827 |  |
|  | *Cryptarcha undata* (Olivier, 1790) | 57 | 593 | 194 | 1 | 844 |  |
|  | *Epuraea aestiva* (Linnaeus, 1758) |  | 1 |  |  | 1 |  |
|  | *Epuraea guttata* (Olivier, 1790) |  | 1 |  |  | 1 |  |
|  | *Epuraea longula (*Erichson, 1845) |  | 1 |  |  | 1 |  |
|  | *Epuraea marseuli (*Reitter, 1872) |  | 1 | 1 |  | 2 |  |
|  | *Epuraea melina (*Erichson, 1843) |  | 4 | 4 |  | 8 |  |
|  | *Epuraea neglecta* (Heer, 1841) |  | 1 |  |  | 1 |  |
|  | *Epuraea pallescens* (Stephens, 1835) |  |  | 1 |  | 1 |  |
|  | *Epuraea rufomarginata* (Stephens, 1830) |  | 1 |  |  | 1 |  |
|  | *Epuraea unicolor* (Olivier, 1790) | 6 | 168 | 193 |  | 367 |  |
|  | *Glischrochilus quadrisignatus* (Say, 1835) | 1 |  |  |  | 1 |  |
|  | *Haptoncus ocularis* (Fairmaire, 1849) | 5 | 63 | 85 | 2 | 155 |  |
|  | *Lamiogethes haemorrhoidalis* (Förster, 1849) | |  |  | 1 | 1 |  |
|  | *Lamiogethes morosus* (Erichson, 1845) | 1 | 1 |  |  | 2 |  |
|  | *Lamiogethes ochropus* (Sturm, 1845) | 1 |  |  |  | 1 |  |
|  | *Omosita discoidea* (Fabricius, 1775) |  |  | 1 |  | 1 |  |
|  | *Soronia grisea* (Linnaeus, 1758) | 2 | 51 | 14 |  | 67 |  |
|  | *Stelidota geminata* (Say, 1825) |  |  | 3 |  | 3 |  |
| Oedemeridae | | 2 | 2 | 1 |  | 5 |  |
|  | *Oedemera femorata* (Scopoli, 1763) | 1 |  |  |  | 1 |  |
|  | *Oedemera podagrariae* (Linnaeus, 1767) | 1 | 2 | 1 |  | 4 |  |
| Ptinidae | | 1 | 4 | 1 | 3 | 9 |  |
|  | *Anitys rubens* (Hoffmann, 1803) |  |  | 1 |  | 1 |  |
|  | *Bruchoptinus rufipes* (Olivier, 1790) |  |  |  | 1 | 1 |  |
|  | *Dorcatoma chrysomelina* (Sturm, 1837) | 1 |  |  |  | 1 |  |
|  | *Gastrallus laevigatus* (Olivier, 1790) |  |  |  | 1 | 1 |  |
|  | *Oligomerus brunneus* (Olivier, 1790) |  | 3 |  |  | 3 |  |
|  | *Ptinus sexpunctatus* (Panzer, 1795) |  | 1 |  | 1 | 2 |  |
| Salpingidae | |  |  | 1 |  | 1 |  |
|  | *Salpingus planirostris* (Fabricius, 1787) |  |  | 1 |  | 1 |  |
| Scarabaeidae | | 2 | 19 | 28 | 3 | 52 |  |
|  | *Amphimallon solstitiale* (Linnaeus, 1758) |  |  |  | 1 | 1 |  |
|  | *Cetonia aurata* (Linnaeus, 1758) |  |  | 1 |  | 1 |  |
|  | *Gnorimus nobilis* (Linnaeus, 1758) |  | 1 |  |  | 1 |  |
|  | *Limarus zenkeri* (Germar, 1813) |  |  |  | 1 | 1 |  |
|  | *Oxythyrea funesta* (Poda, 1761) | 1 | 2 | 1 |  | 4 |  |
|  | *Protaetia cuprea obscura* (Andersch, 1797) | |  | 1 |  | 1 |  |
|  | *Protaetia marmorata* (Fabricius, 1792) |  | 6 | 11 |  | 17 |  |
|  | *Protaetia metallica* (Herbst, 1782) | 1 | 10 | 14 | 1 | 26 |  |
| Scirtidae | | 1 |  |  |  | 1 |  |
|  | *Elodes minutus* (Linnaeus, 1767) | 1 |  |  |  | 1 |  |
| Scraptiidae | | 4 | 4 | 3 | 1 | 12 |  |
|  | *Anaspis brunnipes* (Mulsant, 1856) |  | 2 |  |  | 2 |  |
|  | *Anaspis flava* (Linnaeus, 1758) |  |  | 1 |  | 1 |  |
|  | *Anaspis ruficollis* (Fabricius, 1792) | 2 | 1 | 1 | 1 | 5 |  |
|  | *Anaspis rufilabris* (Gyllenhal, 1827) |  |  | 1 |  | 1 |  |
|  | *Anaspis septentrionalis* Champion, 1891 | 2 |  |  |  | 2 |  |
| Silphidae | |  | 1 | 1 |  | 2 |  |
|  | *Phosphuga atrata* (Linnaeus, 1758) |  |  | 1 |  | 1 |  |
|  | *Thanatophilus sinuatus* (Fabricius, 1775) |  | 1 |  |  | 1 |  |
| Silvanidae | |  |  | 1 |  | 1 |  |
|  | *Ahasverus advena* (Waltl, 1832) |  |  | 1 |  | 1 |  |
| Staphylinidae | | 10 | 59 | 91 | 2 | 162 |  |
|  | *Omalium rivulare* (Paykull, 1789) |  |  | 1 | 1 | 2 |  |
|  | *Phloeostiba plana* (Paykull, 1792 |  |  | 1 |  | 1 |  |
|  | *Quedius cruentus* (Olivier, 1795) | 6 | 15 |  |  | 21 |  |
|  | *Velleius dilatatus* (Fabricius, 1787) | 1 | 11 | 45 |  | 57 |  |
| Tenebrionidae | | 2 | 3 |  |  | 5 |  |
|  | *Cteniopus sulphureus* (Linnaeus, 1758) | 1 |  |  |  | 1 |  |
|  | *Hymenalia rufipes* (Fabricius, 1792) | 1 | 1 |  |  | 2 |  |
|  | *Lagria atripes* (Mulsant, 1855) |  | 1 |  |  | 1 |  |
|  | *Nalassus dermestoides* (Illiger, 1798) |  | 1 |  |  | 1 |  |
| Throscidae | | 2 |  | 3 | 8 | 13 |  |
|  | *Aulonothroscus brevicollis* (Bonvouloir, 1859) | 1 |  | 1 | 7 | 9 |  |
|  | *Trixagus dermestoides* (Linnaeus, 1766) | 1 |  | 2 |  | 3 |  |
|  | *Trixagus meybohmi* (Leseigneur, 2005) |  |  |  | 1 | 1 |  |
| Zopheridae | |  |  | 1 |  | 1 |  |
|  | *Synchita undata* (Guérin-Méneville, 1844) |  |  | 1 |  | 1 |  |
